# Crossmodal Pattern Discrimination in Humans and Robots: A Visuo-Tactile Case Study

**DOI:** 10.3389/frobt.2020.540565

**Published:** 2020-12-23

**Authors:** Focko L. Higgen, Philipp Ruppel, Michael Görner, Matthias Kerzel, Norman Hendrich, Jan Feldheim, Stefan Wermter, Jianwei Zhang, Christian Gerloff

**Affiliations:** ^1^Department of Neurology, University Medical Center Hamburg-Eppendorf, Hamburg, Germany; ^2^Department of Informatics, Universität Hamburg, Hamburg, Germany

**Keywords:** aging, Braille, multisensory integration, neural networks, ANN

## Abstract

The quality of crossmodal perception hinges on two factors: The accuracy of the independent unimodal perception and the ability to integrate information from different sensory systems. In humans, the ability for cognitively demanding crossmodal perception diminishes from young to old age. Here, we propose a new approach to research to which degree the different factors contribute to crossmodal processing and the age-related decline by replicating a medical study on visuo-tactile crossmodal pattern discrimination utilizing state-of-the-art tactile sensing technology and artificial neural networks (ANN). We implemented two ANN models to specifically focus on the relevance of early integration of sensory information during the crossmodal processing stream as a mechanism proposed for efficient processing in the human brain. Applying an adaptive staircase procedure, we approached comparable unimodal classification performance for both modalities in the human participants as well as the ANN. This allowed us to compare crossmodal performance between and within the systems, independent of the underlying unimodal processes. Our data show that unimodal classification accuracies of the tactile sensing technology are comparable to humans. For crossmodal discrimination of the ANN the integration of high-level unimodal features on earlier stages of the crossmodal processing stream shows higher accuracies compared to the late integration of independent unimodal classifications. In comparison to humans, the ANN show higher accuracies than older participants in the unimodal as well as the crossmodal condition, but lower accuracies than younger participants in the crossmodal task. Taken together, we can show that state-of-the-art tactile sensing technology is able to perform a complex tactile recognition task at levels comparable to humans. For crossmodal processing, human inspired early sensory integration seems to improve the performance of artificial neural networks. Still, younger participants seem to employ more efficient crossmodal integration mechanisms than modeled in the proposed ANN. Our work demonstrates how collaborative research in neuroscience and embodied artificial neurocognitive models can help to derive models to inform the design of future neurocomputational architectures.

## Introduction

Human behavior in the natural environment crucially depends on the continuous processing of simultaneous input to different sensory systems. Integration of these sensory streams creates meaningful percepts and allows for fast adaption to changes in our surrounding (Calvert, 2001). The success of this crossmodal integration depends on two factors: The accuracy of the independent unimodal perception and the ability to integrate information from different sensory systems (Calvert et al., 2004).

In a recent human behavioral study (Higgen et al., 2020), we found that older participants show significant difficulties in a well-established visuo-tactile crossmodal discrimination task compared to younger participants (Hummel and Gerloff, 2006; Göschl et al., 2015; Wang et al., 2019). In this task, tactile and visual stimuli are presented simultaneously, representing either the same or different geometrical dot patterns. Participants have to decide whether the patterns were congruent or incongruent. We applied an adaptive staircase procedure prior to the crossmodal task, to determine unimodal classification thresholds. This approach allowed us to investigate crossmodal performances at stimulus intensities with comparable unimodal classification accuracies.

With aging, performance decreases in several cognitive processes (Gazzaley et al., 2005; Anguera and Gazzaley, 2012; Heise et al., 2013; Guerreiro et al., 2014). The processing of unimodal sensory stimuli constitutes one major domain of this deterioration (Freiherr et al., 2013). However, our data revealed that difficulties of older participants go beyond a simple decline in unimodal stimulus classification, i.e., the identification of a stimulus presented in one modality. The data suggest that the crossmodal integration of information from different sensory systems in higher-order neural networks might be one of the key reasons of poor performance of older participants. As the percentage of older people in the overall population increases, age-related declines gain more and more importance. Understanding the mechanisms of these declines is vital to develop adequate support approaches (see for example Krawinkel et al., 2015). However, age-related alterations in human neural networks and their effects on local computing and long-range communication in the brain, which are needed for crossmodal integration, are not well understood (Hong and Rebec, 2012; Schulz et al., 2014; Quandt et al., 2016). It has been suggested to understand the aging of the brain as a network level phenomenon, leading for example to decreased structural and functional connectivity in whole-brain networks (Geerligs et al., 2015; Sala-Llonch et al., 2015; Damoiseaux, 2017; Zonneveld et al., 2019). However, causal assignment of altered neural function or network properties to behavioral changes is one of the great challenges in neuroscience.

In the current study, we propose a new approach to research the relevance of different network properties in crossmodal integration by adapting our recent human behavioral study to an artificial neural network (ANN) scenario. On the one hand, network models might help to understand the reasons for poor performance in older humans. One the other hand, the design of high-performing artificial neural networks for crossmodal integration is likewise one of the most significant challenges in robotics (see for example Ngiam et al., 2011; Feng et al., 2015; Guo et al., 2019; Müller-Eberstein and van Noord, 2019; Wang et al., 2020). We suggest, that adaption of human inspired mechanisms and comparison to human performance will allow for an evaluation of the performance of artificial systems compared to humans with different abilities and help to develop more biologically plausible and performant artificial neural network models (Barros and Wermter, 2016; Deistler et al., 2019; Fu et al., 2020).

Specifically, we replicated our human behavioral study on visuo-tactile crossmodal pattern discrimination utilizing state-of-the-art tactile sensing technology and artificial neural networks. We employed embodied neurocognitive models to evaluate a specific hypothesis of the contribution of unimodal processing and crossmodal integration to the visuo-tactile discrimination task. Classical studies have postulated crossmodal integration to be achieved by hierarchical convergence of unimodal information onto specialized multisensory brain regions (Stein and Meredith, 1993; Meredith, 2002). More recent work, however, highlights the importance of distributed multisensory processing and the integration of information already on earlier stages of the processing stream such as primary sensory cortices (Zhou and Fuster, 2000; Ghazanfar and Schroeder, 2006; Kayser and Logothetis, 2007; Senkowski et al., 2008). To evaluate whether this early integration of crossmodal information might be one mechanism relevant for high performance in the visuo-tactile discrimination task we implemented two ANN models. The first artificial network (V-architecture) implements a model for the late integration of fully processed results of the unimodal sensory streams. In contrast, the second network (Y-architecture) implements a model with an emphasis on the early integration of information during crossmodal processing, integrating complex higher-level features from the unimodal streams. Importantly, the unimodal processing streams of both networks were identical in terms of architecture.

To establish comparability between the human participants and the ANN and to account for confounding factors such as visual acuity of the human participants or hardware specifics of the ANN, we made the effort to adapt the staircase procedure from the human behavioral study to the artificial scenario. After training of the unimodal pattern classification, we estimated stimulus intensities at which the performance of the unimodal processing streams matched the performance of the human participants at their unimodal classification thresholds. Thus, we achieved comparable unimodal pattern classification performance for both modalities in the human participants as well as the ANN. This allowed us to compare the crossmodal performance of the different artificial neural networks and the human participants, independent of the underlying unimodal processes.

Due to the limited complexity of the artificial neural networks, we hypothesize that the younger human participants will show best crossmodal discrimination performance. Furthermore, we hypothesize that the human inspired early integration of crossmodal information will outperform the late integration artificial neural network model.

## Materials and Methods

### Visuo-Tactile Discrimination Task in Humans

**Figure 1:**
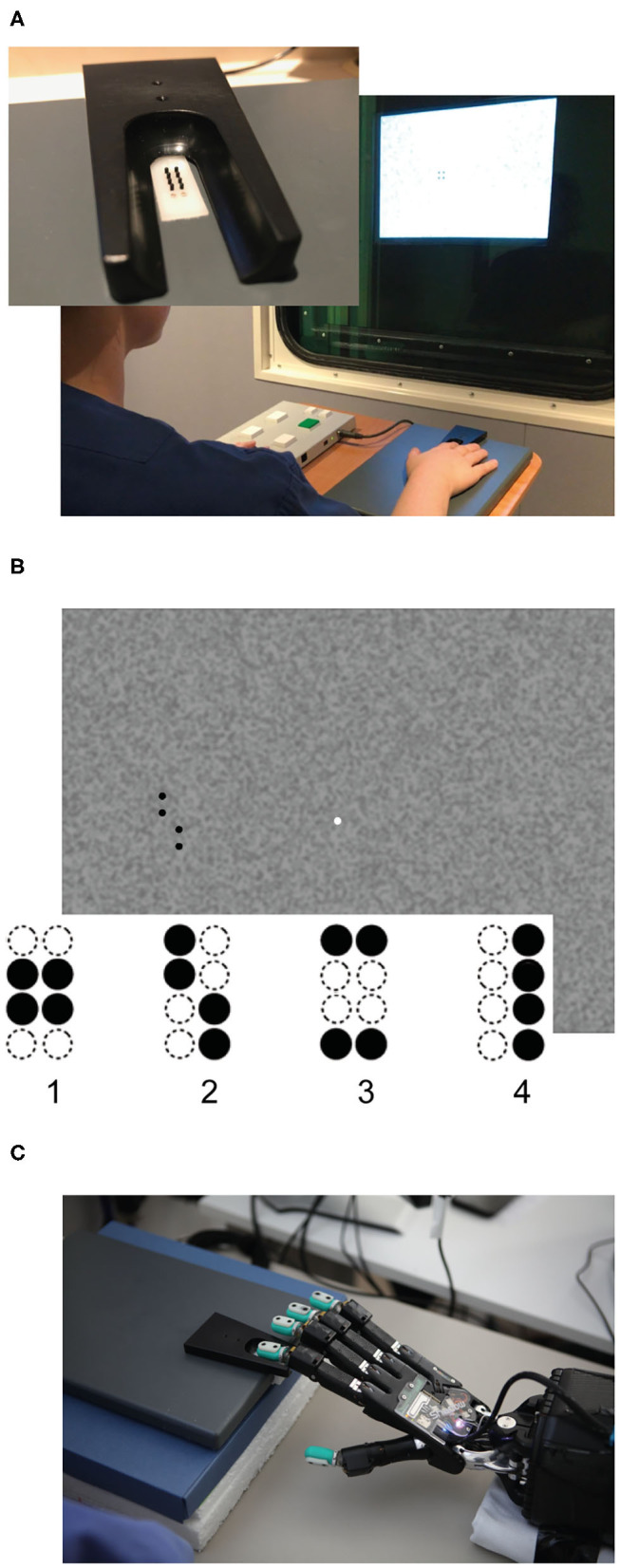
Experimental setup. **(A)** Braille stimulator and setup of the human behavioral experiment. For tactile stimulation, the participants' right hand was resting on a custom-made board containing the Braille stimulator (QuaeroSys Medical Devices, Schotten, Germany), with the fingertip of the right index finger placed above the stimulating unit. Visual patterns were presented on a monitor and the participants indicated whether both patterns were congruent or incongruent. The task was rendered more difficult by blending the visual pattern in the background noise and by reducing the actuated pin height. **(B)** One example input for visual patterns with 100% intensity (i.e., full black). In both experiments, stimuli consisted of the same four patterns (1–4). **(C)** Setup of robot experiment using a Shadow C6 Dexterous Hand. The BioTac tactile fingertip of the first finger of the hand is placed on the Braille stimulator.

In our human behavioral experiment, 20 younger (11 female, *M* = 24.05 years, SD = 2.50) and 20 healthy older volunteers (11 female, *M* = 72.14 years, SD = 4.48) performed an adapted version of a well-established visuo-tactile pattern discrimination task (Hummel and Gerloff, 2006; Göschl et al., 2015; Wang et al., 2019; Higgen et al., 2020). In this task, participants had to compare Braille patterns presented tactilely to the right index fingertip with visual patterns presented on a computer screen (Figure 1). Patterns appeared simultaneously with synchronous onset and offset and were either congruent (i.e., represented the same geometrical pattern) or incongruent (i.e., represented different geometrical patterns). Participants had to indicate whether patterns were congruent or incongruent. Importantly, the task did not explicitly require a classification of the presented patterns. Tactile stimulation was delivered via a Braille stimulator (QuaeroSys Medical Devices, Schotten, Germany, see Figure 1A), consisting of eight pins arranged in a four-by-two matrix, each 1 mm in diameter with a spacing of 2.5 mm. Each pin is controlled separately. Pins can be elevated (maximum amplitude 1.5 mm) for a specific duration to form different patterns. Visual patterns were designed analogously to the Braille patterns and presented left of a central fixation point on a noisy background (Perlin noise; see Figure 1B). The visual patterns subtended 3.5° × 2.5° of visual angle. A set of four clearly distinct patterns was used in the study (see Figure 1B) to account for the diminished unimodal tactile perception of the older participants. None of the participants had prior experience with reading Braille.

To be able to compare crossmodal performance of the human younger and older participants, we aimed to achieve a comparable performance of around 80% correct answers for both unimodal classification tasks. To this end, prior to the crossmodal task, each participant performed a unimodal adaptive staircase procedure with a target classification accuracy of approximately 80% to individually adjust stimulus intensities in the visual and tactile modalities (Wetherill and Levitt, 1965; Kaernbach, 1991; Treutwein, 1995; García-Pérez, 1998). The adaptive-staircase procedure was performed in both modalities separately, to ensure comparable unimodal classification performance across modalities and between older and younger participants. Tactile stimulus intensity was adjusted by changing the height of the braille pattern (pin height). Visual stimulus intensity was adjusted by changing the patterns' contrast against the background (gray level in % of black). Pilot data showed that a gray intensity of 47% of black was hardest to detect on the Perlin noise background. Therefore, this contrast was the lower boundary of the staircase, with the upper boundary being 100% of black.

Finally, participants performed the visuo-tactile discrimination task at the afore-defined unimodal thresholds. Furthermore, the same group of younger participants performed the task at thresholds comparable to the older group (=younger controls). These thresholds were estimated based on piloting and did not differ significantly from the mean of the individual thresholds of the older group. The control group allowed us to compare crossmodal performance of younger and older participants at the same stimulus intensities.

The study was conducted in accordance with the Declaration of Helsinki and was approved by the local ethics committee of the Medical Association of Hamburg. All participants gave written informed consent. For a detailed description of the experiment, please see Higgen et al. (2020).

### Robotic Adaption

The setup described above was implemented in a robotic experiment (Figure 1C).

For tactile stimulation, the same Braille stimulator (QuaeroSys Medical Devices, Schotten, Germany, see Figure 1A) and the same set of Braille patterns were used (Figure 1). The tactile stimuli were applied to the fingertips of a Shadow C6 Dexterous Hand^1^ equipped with BioTac tactile sensors.^2^ The sensor surface of the BioTac closely matches the size and shape of a human finger, and it was possible to align and center the sensor onto the Braille stimulator without modifying the setup. To perceive the tactile stimuli of the Braille stimulator, the sensor can detect multiple contacts through indirect measurement. The turquoise rubber shell is filled with a conductive liquid and held in place around an inner rigid “bone.” When contacting an object, the rubber deforms, changing the overall pressure of the liquid (1 channel) and also the impedance between a set of electrodes patterned on the bone (19 channels). At the same time, the liquid temperature changes due to the contact (2 channels). Raw data from the sensor combines the measured pressure, temperature and impedances but it is difficult to interpret this raw data (Chia-Hsien et al., 2009; Wettels, 2011; Lin and Loeb, 2013). Because the temperature only changes slowly and does not immediately react to stimulation from the Braille simulator, we omitted the respective sensor readings and fed the remaining 20 channels into an artificial neural network (ANN) to learn the mapping from raw data to applied Braille pattern. The sensor produces a continuous stream of tactile data. For each stimulation, we fed a sequence of 150 samples from shortly before, during and after the stimulation into the network. The number of samples collected during each stimulus is roughly equal to the numbers before and after the stimulus. In line with our human behavioral experiment, tactile stimulus intensity was adjusted by changing the height of the braille pattern (pin height), with the maximum amplitude being 1.5 mm.

**Figure 2:**
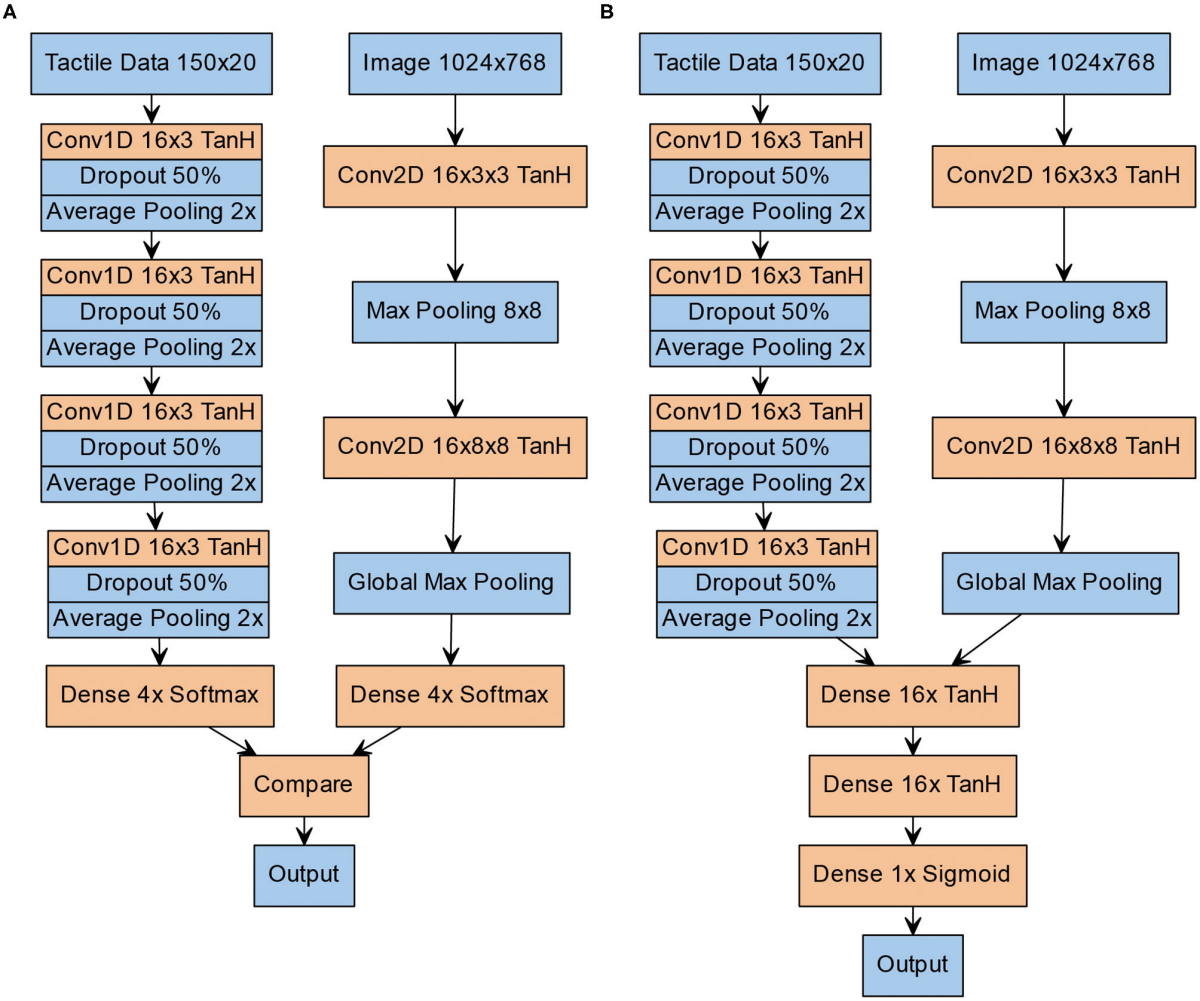
Structure of the neural architectures. **(A)** Structure of the V-architecture. Visual (left column) and tactile data (right column) are processed separately and statically compared in the end. **(B)** Structure of the Y-architecture. Both columns are first trained separately on visual and tactile data. Afterwards, a number of densely connected layers and a sigmoid output layer are added, and the network is trained again on the combined visual and tactile data.

As visual stimuli, we used the same visual stimuli employed in the human experiment (Figure 1). These stimuli were directly fed into the neural architecture without an intermediate sensor like a camera. As detailed below, the comparison with the human experiments relies on the exact gray values used in the stimuli; direct input of the images to the ANN avoids any level-shifts due to inconsistent camera exposure control. The noisy visual input images are generated by placing one of four target Braille patterns (43 × 104px) randomly on one of 48 randomly generated background images (1024 × 768px, see Figure 2 left). The background consists of a Perlin noise pattern with a gray range of between 40 and 60% of black (mean 53.7%). In line with our human behavioral experiment, the stimulus intensity (i.e., gray level in % of black) of the pattern was selected to be between 47 and 100%. Samples were dynamically generated for each episode.

As the classification of the tactile and visual stimuli require offline learning, the adaptive staircase procedure used in the human behavioral experiment could not be adapted to our machine learning approach. However, to ensure comparability, we employed a methodology that mimics the adaptive staircase procedure. In the visual as well as in the tactile modality we recorded datasets with different stimulus intensities, i.e., datasets for all gray levels in the visual modality and datasets over the whole range of pin heights in the tactile modality. To this end, we recorded several hours of raw sensor data from the robot, labeled with the presented tactile or visual patterns, respectively. In total, 3,000 tactile samples were collected to obtain a sufficient number of haptic samples with different pin heights. Each of the datasets was used to train a unimodal neural classifier. The training for each classifier was optimized with regard to the dataset. For example, the duration of the training and the number of training iterations was empirically determined for each dataset to yield the highest possible classification accuracy. Depending on the stimulus intensity (pin height or gray level) and the unimodal network different classification results can be achieved for the unimodal classification tasks.

Based on these results in the unimodal condition, in the crossmodal task we could present visual and tactile stimulus intensities to the trained ANN that achieved a unimodal classification accuracy of about 80% correct, in correspondence with the human behavioral approach. In the crossmodal task, again in line with our human behavioral experiment, visual and tactile stimuli were paired so that the probability of both stimuli within a crossmodal sample pair representing the same pattern was 50%, equal to the probability of both stimuli representing different patterns. All test results for the ANN in the crossmodal task were obtained through 10-fold cross validation. Two-sampled *t*-tests were used to compare performance of the Y- and V-architecture in the crossmodal task over the whole range of stimulus intensities as well as for selected stimulus pairs.

### Computational Models

The tactile and the visual information are input into ANN that output if the patterns in both modalities are congruent or incongruent, corresponding to the task demands in the human behavioral study. To evaluate the influence of the integration of high-level unimodal features on earlier stages of the crossmodal processing stream in contrast to just comparing unimodal classifications, we propose two neural architectures: (a) the V-architecture (see Figure 2A) performs two separate unimodal pattern classifications that determine which of the four Braille patterns was input to the tactile and the visual modality before these results are compared; and (b) the Y-architecture (see Figure 2B) performs an early integration of the crossmodal information by integrating high-level feature representations of both modalities. Importantly, unimodal processing streams of both networks are identical in terms of architecture. The development of both architectures was informed by previous work. Due to the high computational cost of the training process, we restricted the automated hyperparameter search to a small set of promising candidate architectures.

The V-architecture can be seen as consisting of two separate networks that perform unimodal classification of the tactile and visual input pattern. For the tactile modality, we use a one-dimensional convolutional neural network with 16 channels in each layer, a kernel size of 3 and TanH activation. After each convolutional layer, we add 50% dropout and average pooling. Local average pooling with a width of 2 is used between the convolutional layers and global average pooling is used after the last convolutional layer. For the visual modality, we use two 16-channel convolutional layers with a size of 3 × 3 in the input layer and 8 × 8 in the hidden layer. Between both convolutional layers, we add 8 × 8 max pooling and after the second convolutional layer, we apply global max pooling. The final layer of each of these subnetworks is a soft-max layer with 4 units, corresponding to the 4 Braille patterns. The two outputs of both soft-max-layers are compared for equality in the final layer by a static (non-learned) operation.

In contrast, the Y-architecture integrates high-level feature representations of both modalities. Like the V-architecture it has two separate columns for unimodal feature extraction from the visual and tactile data that are identical in design and hyperparameters to the arms of the V-architecture. However, instead of individual softmax-layers the features extracted by the unimodal arms of the network are concatenated and further integrated in the stem of the Y-architecture by two dense layers with 16 neurons each followed by a sigmoid layer that outputs an answer to the question if the crossmodal inputs represent the same pattern, i.e., if they are congruent.

### Unimodal and Crossmodal Training

The training for both networks was supervised and followed the same pattern: First, each unimodal column of the network was pre-trained. The tactile branch was pre-trained for 500 episodes and the visual network for 10 episodes using the above described dataset with a randomized 90/10 split into training and test data with a ten-fold cross validation. Due to the relatively small size of the dataset, no validation set for early stopping was employed, instead the number of training epochs was determined empirically. For the V-architecture, the static comparator required no further training. For the crossmodally integrating Y-architecture, a second training phase followed where the crossmodal layers of the Y-architecture were trained: The weighs of the pre-trained unimodal network arms were frozen and the stem of the Y-network was trained for 5 epochs with randomly combined visuo-tactile data consisting of 5 times more samples than the original tactile dataset. The method of pretraining the unimodal arms of the architecture and then freezing their weights before training the complete crossmodal architecture was chosen to prevent destructive interference between the different modalities (Zhao et al., 2018). For all training phases, the Adam optimizer with a learning rate of 0.001 was used. Categorial crossentropy was used as a loss function. The images were not preprocessed before training or testing. They were fed as raw RGB images (1024 × 768 pixels) into the network. Likewise, the tactile data were fed as 150 × 20 matrices into their respective network. The hyperparameters of the architecture in terms of layer sizes and activation functions are depicted in more detail in Figure 2. For training the crossmodal Y-architecture and for pre-training the visual column, a batch size of 16 was used. The tactile column was pre-trained with a batch size of 1,024. Again, these hyperparameters, including the choice of the optimization algorithm, were determined based on previous work and empirical exploration of candidate parameters.

## Results

### Human Behavior

In our human behavioral study, unimodal tactile and visual thresholds for a pattern classification accuracy of around 80% were estimated by implementing an adaptive staircase procedure (see Table 1). Older participants showed higher thresholds for unimodal tactile and visual pattern classification compared to younger participants. In the crossmodal task older participants showed a significantly weaker performance compared to the unimodal condition when using the individual unimodal perception thresholds. In contrast, younger participants showed a stable performance of around 80% (see Table 1).

**Table 1:** Performance of older and younger participants in the visuo-tactile discrimination task.

	**Unimodal tactile threshold (pin height in μm)**	**Unimodal visual threshold (gray level in %)**	**Accuracy in the crossmodal task at unimodal thresholds (%)**
Older participants	1,143.0	53.65	66.2
Younger participants	576.8	49	78.31
Younger controls	1,355.3	57	96.2

*Unimodal tactile (Braille pin height in μm) and visual threshold (gray level in % of black) for 80% classification accuracy, and performance on the visuo-tactile discrimination task at the unimodal thresholds, for younger and older human participants. Younger participants show a stable crossmodal performance of around 80% classification accuracy at their individual unimodal thresholds. Classification accuracy <80% in the older group implies a degradation of crossmodal integration*.

In a control experiment, the same group of younger participants (=younger controls) showed a performance of 96.2% in the visuo-tactile discrimination task at thresholds comparable to the older group.

### Artificial Neural Networks

**Figure 3:**
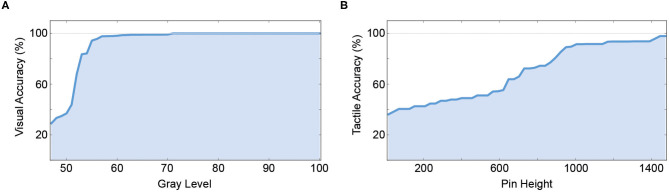
Unimodal performance of the artificial neural network. **(A)** Unimodal performance of the visual pattern classification network which is also used as the visual branch in the V- and Y-architectures by gray level (% of black). **(B)** Unimodal performance of the tactile pattern classification network which is also used as the tactile branch in the V- and Y- architectures by pin height (in μm).

To test the individual classification accuracy of both unimodal channels, and to compare them to the performance of the human participants in the original experiment, both models were fed inputs of varying difficulty (gray level for the visual channel, pin height for the haptic channel). A 10-fold cross-validation was performed by splitting the 3,000 samples into 90% training and 10% test data. The results for the unimodal visual and tactile channel can be seen in Figure 3.

In the unimodal tactile condition, we were able successfully classify the Braille patterns from the raw BioTac sensor data. The artificial network showed a sigmoid learning curve, comparable to the human participants. At pin heights of 1,445 μm and above a classification accuracy of 98% was reached. A classification accuracy of 80% was reached around 895 μm (81% accuracy). In the unimodal visual condition, the classification accuracy was high (on average 92.31%). The classification accuracy was at 100% correct for a wide range of gray values and started dropping once the gray value of the pattern also appeared in the background image (values between 40 and 60%). A classification accuracy of 80% was reached at a gray level of 53 (83.4% accuracy).

**Figure 4:**
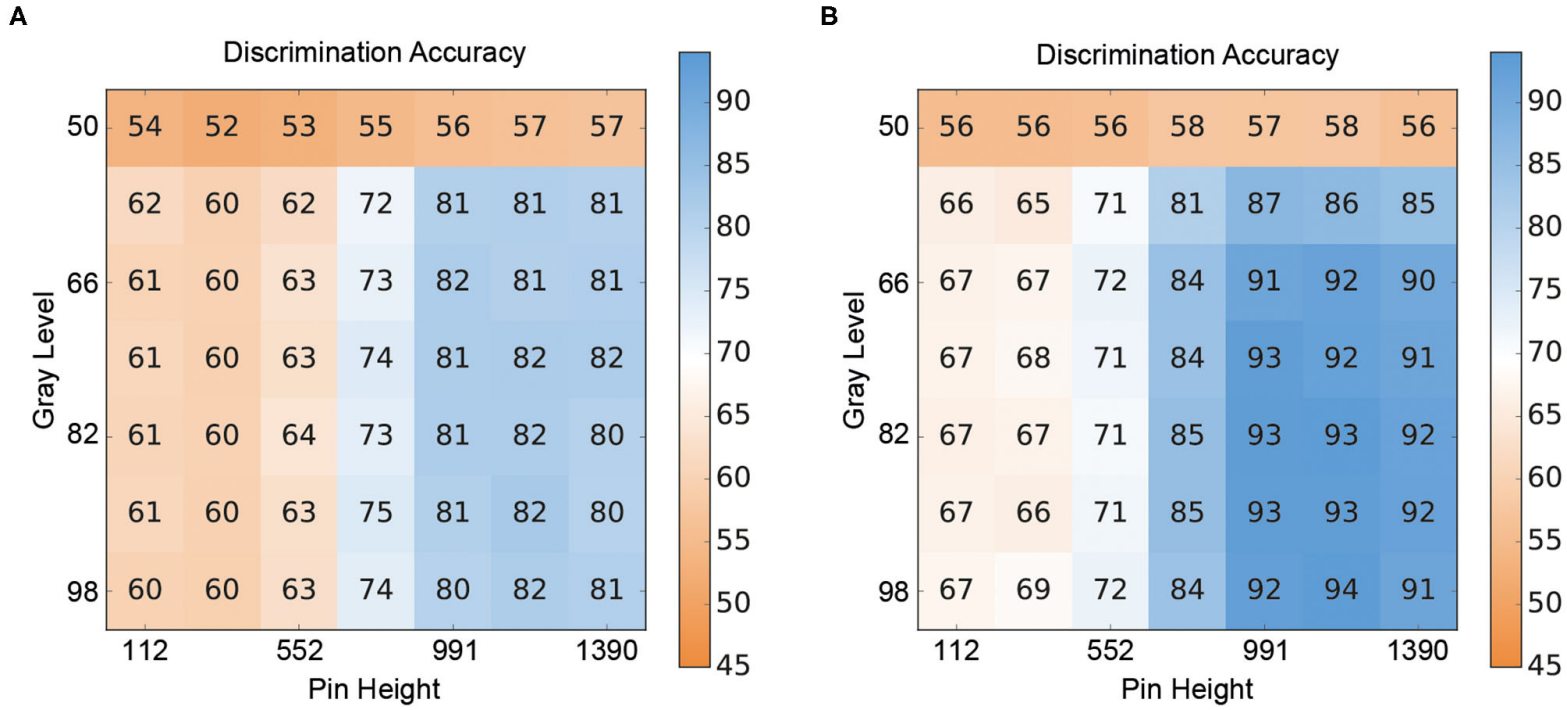
Crossmodal performance of the neural architectures. **(A)** Performance of the V-architecture. Discrimination accuracy of the V-architecture (in %) by pin height and gray level. Parameters are the gray level (% of black) of the visual pattern and the active pin height (μm) of the Braille stimulator. (**B)** Performance of the Y-architecture. Discrimination accuracy of the Y-architecture (in %) by pin height and gray level. Parameters are the gray level (% of black) of the visual pattern and the active pin height (μm) of the Braille stimulator.

The results for the crossmodal visuo-tactile discrimination task are shown in Figure 4. Figure 4 depicts the discrimination accuracies (i.e., correct discrimination whether the presented patterns were congruent or incongruent) of the two ANN for different combinations of stimulus intensities. Figure 4A shows the results for the V-architecture. As expected, the performance of the network degraded when the channels were too noisy, but accuracy improved quickly as the signal quality (gray level, pin height) became better. The corresponding results for the Y-architecture (Figure 4B) showed a similar structure, but with higher discrimination accuracies over the whole range of stimulus intensity pairs (*t* = 4.69, *p* < 0.001).

When comparing the performance of the two ANN in the crossmodal task at the 80% classification thresholds determined for the unimodal branches, both ANN showed a decrease in performance compared to the unimodal condition (see Table 2). However, the Y-architecture showed higher accuracies at these stimulus intensities (75.3%) compared to the V-architecture (69.2%). In comparison, younger human participants showed best performance close to 80% correct (78.31%) at their individual unimodal thresholds, while older participants showed an even larger decrease in accuracies in the crossmodal task (66.2%; see Table 1).

**Table 2:** Performance of the artificial neural networks at signal levels comparable to the human participants.

	**Pin height (μm)**	**Gray level (%)**	**V-architecture: Accuracy in the crossmodal task (%)**	**Y-architecture: Accuracy in the crossmodal task (%)**
Signal levels comparable to older participants	1,143	54	73.7	79.1
Signal levels comparable to younger participants	593	49	48.8	55.0
Unimodal thresholds of the ANN	895	53	69.2	75.3

*Discrimination accuracy of the artificial networks at tactile (Braille pin height in μm) and visual (gray level in % of black) signal levels comparable to the thresholds determined in the human experiments and thresholds determined for the unimodal artificial neural networks (ANN) branches*.

Evaluation of the crossmodal performance of the ANN at the unimodal thresholds determined for the human participants, showed that both networks perform better than the older participants (66.2%) at the unimodal thresholds of the older group (V-architecture: 73.7%; Y-architecture: 79.1%). Compared to the younger group (78.31%) both networks showed poorer performances at the unimodal thresholds of the younger group (V-architecture: 48.8%; Y-architecture: 55.0%).

**Figure 5:**
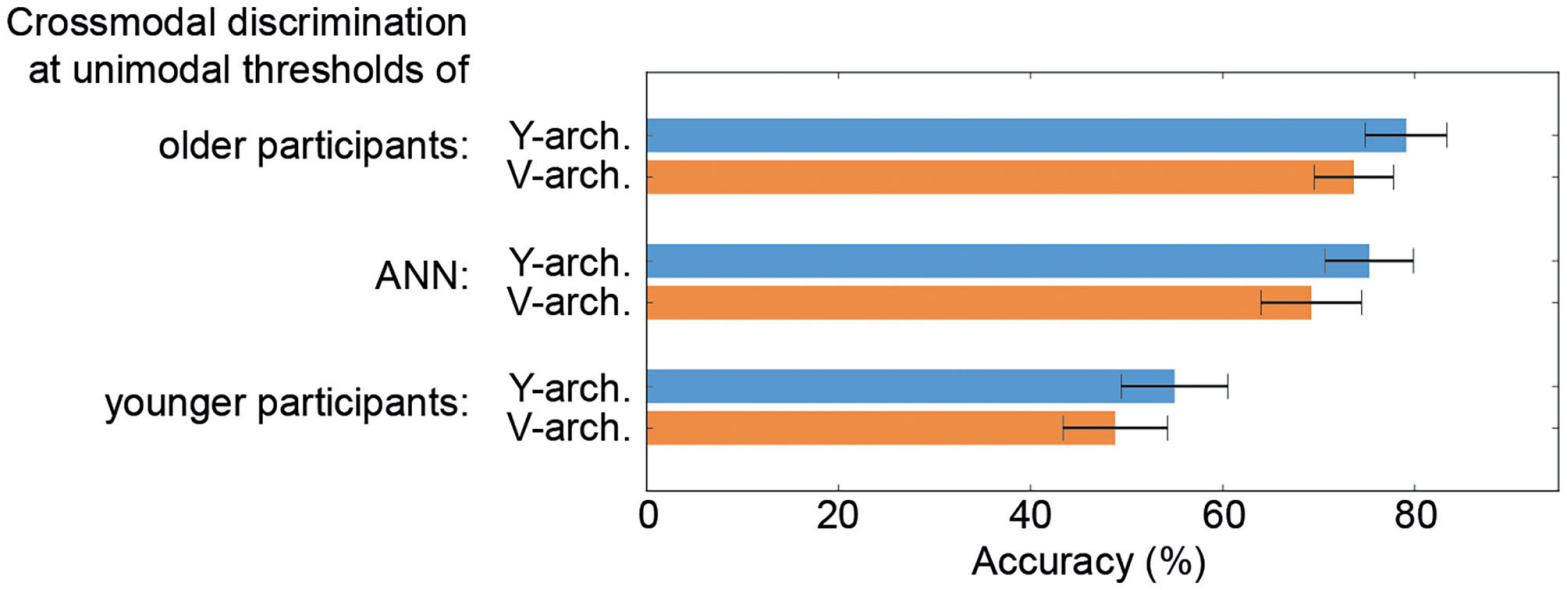
Performance comparison of the neural architectures. Results from a 10-fold cross validation to obtain average crossmodal discrimination accuracies with standard error confidence measures for the V- and Y-architecture at unimodal thresholds of the older (top) and younger participants (bottom) as well as for the thresholds determined for the unimodal artificial neural networks (ANN) branches (middle) are depicted.

When comparing the performances of the two ANN at these selected stimulus intensity pairs (unimodal thresholds of the older and younger participants as well as thresholds determined for the unimodal ANN branches) the Y-architecture shows numerically higher performance for all stimulus pairs, even though the differences are not significant (all *p*-values > 0.05; see Figure 5).

## Discussion

In this study, we propose a novel approach for collaborative neuroscientific and robotic research to better understand and apply mechanisms of crossmodal integration. We aimed to investigate the transfer of a human behavioral experiment on crossmodal visuo-tactile pattern discrimination to an artificial neural network scenario and to compare the performance of different embodied neurocognitive models to the performance of younger and older humans.

For unimodal tactile pattern classification, we used state-of-the-art sensing technology to learn the mapping from raw data to applied Braille patterns. For visuo-tactile pattern discrimination we implemented two artificial neural network models to evaluate the relevance of early integration of sensory information as a mechanism for crossmodal processing. The first artificial network (V-architecture) implements a model for the late integration of fully processed results of the unimodal sensory streams. In contrast, the second network (Y-architecture) implements a model with an emphasis on the integration of information during earlier stages of the crossmodal processing stream, integrating complex higher-level features from the unimodal streams. We made use of an adaptive staircase procedure, to approach comparable unimodal pattern classification accuracies for both modalities in the human participants as well as the ANN. This allowed us to compare crossmodal performances, independent of the respective unimodal classification capabilities.

The data show that in an artificial system, early integration of complex high-level unimodal features outperforms the comparison of independent unimodal classifications. Importantly, their unimodal processing columns were identical in terms of architecture. In our corresponding human behavioral experiment younger participants showed a stable performance in the crossmodal task at the unimodal thresholds while older participants showed a significantly weaker performance (Higgen et al., 2020). In line with previous data, the results suggested altered mechanisms of crossmodal integration in the aged brain (Mozolic et al., 2012; Freiherr et al., 2013; de Dieuleveult et al., 2017). Intriguingly, both datasets indicate that not only classification of unimodal stimuli but also mechanisms of integration are crucial for performance in crossmodal integration. The data of our two corresponding experiments now allow for comparing performances of the artificial neural networks and the human participants.

In both modalities, we reached a unimodal classification accuracy of the ANN of around 100% (visual 100% tactile 98%) correct. This performance was reached with maximum pin height in the tactile condition and a wide range of gray intensities in the visual condition. For both modalities, we could determine stimulus intensities with a classification accuracy of the unimodal artificial neural network branches of around 80% correct. In the visual as well as in the tactile modality, performance of the ANN lies between the human younger and older participants. In the visual condition performance is closer to the older participants. However, comparing this performance to the human participants is difficult, as stimuli were directly fed into the neural architecture without an intermediate sensor like a camera. Real-world data collection with adaptive camera exposure might lead to a weaker performance of the ANN in visual pattern classification. In the unimodal tactile condition, the experimental setup in the robotic adaption was exactly the same as in the human behavioral experiment. The performance in unimodal tactile pattern classification of the ANN lies just in between the younger and the older participants. The results show that state-of-the-art sensing technology can perform a complex pattern classification task at a level almost comparable to young humans (Dsouza, 2016). Moreover, the artificial networks perform distinctively better than the older human participants.

In the crossmodal discrimination task, the Y-architecture shows significantly better performance over the whole range of stimulus intensity pairs compared to the V-architecture. The performance of both networks is better compared the older participants at the unimodal thresholds of the older group and worse compared the younger participants at the unimodal thresholds of the younger group. These performance differences are most likely due to the differences in unimodal pattern classification. The most interesting is the comparison of the crossmodal performance at the individual unimodal thresholds between groups. At these stimulus intensities, unimodal classification accuracies are comparable between human younger and older participants as well as the ANN and should, therefore, not account for any differences in crossmodal performance. The performance at these stimulus intensities might be seen as an indicator for the efficacy of the mechanisms of crossmodal integration. The younger participants show a stable performance in the crossmodal task at their individual unimodal thresholds (78.31%), while older participants show a distinct decrease in performance (66.2%). For the crossmodal performance of the ANN at the thresholds determined for the unimodal branches, we find that the V-architecture shows a decrease in performance almost comparable to the older group (69.2%). The Y- architecture shows a slight decrease in performance as well, however, performance is still above 75% (75.3%). The high performance of the Y-architecture is further corroborated by reaching a discrimination accuracy of 94% correct for high stimulus intensities, compared to an accuracy of 81% correct for the V-architecture. Despite the numerically better performance of the Y-architecture compared to the V-architecture, statistical analysis did not reveal a significant difference. We find a better performance of the Y-architecture for all selected stimulus intensity pairs (see Figure 5), which reflects the overall significantly better performance. However, comparison of the performance for single stimulus pairs did not reveal significant results. This is most likely due to the small sample size obtained from the 10-fold cross validation. We do not attribute the better performance of the early integrating architecture merely to the additional model parameters introduced by the integration layer. The unimodal architectures are optimized with regard to the number of parameters (represented by the number of neurons in the architecture). Empirical results have shown that more parameters do not lead to better, but worse results, as the architecture tends to overfit on the training data. In contrast, we suggest an alternative explanation: If two monomodal classifications are performed, and only the final results of the two classifications are compared, any information on the distribution over the possible classification outcomes except the winning outcomes is lost.

Taken together, our data indicate that mechanisms of crossmodal integration are most efficient in the younger human participants compared to the older participants but also the artificial neural networks. Younger participants show best performance at their individual unimodal classification thresholds. However, the Y-architecture seems to approach the crossmodal performance of the younger participants. The Y-architecture is more efficient compared to the V-architecture in integrating crossmodal stimuli over the whole range of stimulus intensity pairs and shows higher performance values at the unimodal classification thresholds. Older participants show weakest performance at their individual unimodal thresholds, indicating a decline of mechanisms of crossmodal integration.

In conclusion, the results for the artificial neural networks as well as the human participants emphasize the importance of the mechanisms of integration for successful crossmodal performance. Integration of incompletely processed sensory information during early stages of the crossmodal processing stream seems to be crucial for efficient crossmodal integration (Molholm et al., 2002; Kayser and Logothetis, 2007; Kayser et al., 2008; Stein and Stanford, 2008). One might argue, that compared to the late integration model of the V-architecture the Y-architecture represents a more biological plausible network, approaching the efficient crossmodal processing in the young human brain. Late convergence of unimodal information does not seem to be suitable to depict the processes in the human brain. Accordingly, a decline in crossmodal integration processes is accompanied with poorer performance, as shown for the older participants in the human behavioral study. Still, our results suggest a superior mechanism for crossmodal stimulus processing in the young human brain compared to the artificial neural networks. Further research is needed to answer the question of how young brains successfully integrate crossmodal information and which of these mechanisms can be adapted in artificial systems. It has been suggested that efficient stimulus processing in the human brain depends on recurrent neural networks and sensory integration on even lower levels (Foxe and Schroeder, 2005; Ghazanfar and Schroeder, 2006). Developing such approaches in future work might, on the one hand, improve the performance of artificial devices, but on the other hand, also give insights into the question which disturbances of the system lead to suboptimal functioning in the aged brain.

There are some limitations to the current study. Comparison of the human and the robotic performance is complicated as no statistical comparison between these groups is possible. Still, our approach allows for indicative comparison between the different systems and, consecutively, statistical within group comparison of the different network models proposed. Furthermore, comparability of the artificial neural network models to mechanisms in the human brain is limited, as they cannot replicate the complexity of human neural networks. Likewise, biological systems (at the time of data collection) are already exposed to many years of continuous and diverse learning, while neuro robotic models are usually trained from scratch with the limited number of samples that are collected in a very specific setup. This difference in training samples has to be reflected in the complexity of the neuro robotic models: in comparison to biological architectures, the neurorobotic architectures need to have a limited number of parameters to prevent overfitting on the training data. In turn, this limitation also limits the architectural complexity of the models. When designing the architectures presented in this paper, best practices in neurorobotics were followed, i.e., different architecture variants were empirically optimized and evaluated with the available training and test data. The resulting architectures reflect the best setup for the data: complex enough to solve the problem but simplistic enough to not overfit to the training data (and fail on the test data). It can be assumed that the optimal architectures would grow in complexity and parameter number with more and more diverse training data.

However, despite the simplistic nature of the neuro robotic architectures, we can show that integrating complex higher-level features from the unimodal streams yields better results for the crossmodal discrimination task than only comparing the fully processed results of the unimodal streams, giving evidence for the advantage of earlier integration. Finally, we were able to demonstrate the adaption of a human neuroscientific experiment to a robotic scenario and to address one specific question regarding the processing of crossmodal information in neural networks. We propose this collaborative approach to better understand and apply mechanisms of crossmodal integration. Likewise, this approach can be used in very different scenarios to establish common grounds in human and robotic research for the mutual exchange of theory (Brooks and Stein, 1995; Hinz et al., 2018; Lanillos et al., 2020). Joint experiments in human and robotic research might help to generate hypotheses for further explorations and to address detailed research questions. For instance, the effects of recurrent neural networks on the performance in crossmodal integration could be compared to the classically used feedforward artificial network models.

## Data Availability Statement

The raw data supporting the conclusions of this article will be made available by the authors, without undue reservation, to any qualified researcher.

## Ethics Statement

The studies involving human participants were reviewed and approved by Medical Association of Hamburg, Weidestraße 122B, 22083 Hamburg, Germany. The participants provided their written informed consent to participate in this study. Written informed consent was obtained from the individual(s) for the publication of any potentially identifiable images or data included in this article.

## Author Contributions

FH, PR, and MG: study idea, study design, data acquisition, data analyses, interpretation, and preparation of manuscript. MK: study idea, interpretation, and preparation of manuscript. NH: study idea, study design, interpretation, and preparation of manuscript. JF: study design, interpretation, and revision of manuscript. SW and JZ: interpretation and revision of manuscript. CG: study idea, interpretation, and revision of manuscript. All authors contributed to the article and approved the submitted version.

## Conflict of Interest

The authors declare that the research was conducted in the absence of any commercial or financial relationships that could be construed as a potential conflict of interest.

## Footnotes

^1^(online: www.shadowrobot.com).

^2^SynTouch LLC, BioTac Product Manual (V21), SynTouch LLC, California, August 2018. https://www.syntouchinc.com/wp-content/uploads/2018/08/BioTac-Manual-V.21.pdf (29).
